# Proteomic Biomarkers for the Prediction of Transition to Psychosis in Individuals at Clinical High Risk: A Multi-cohort Model Development Study

**DOI:** 10.1093/schbul/sbad184

**Published:** 2024-01-19

**Authors:** Jonah F Byrne, Colm Healy, Melanie Föcking, Subash Raj Susai, David Mongan, Kieran Wynne, Eleftheria Kodosaki, Meike Heurich, Lieuwe de Haan, Ian B Hickie, Stefan Smesny, Andrew Thompson, Connie Markulev, Alison Ruth Young, Miriam R Schäfer, Anita Riecher-Rössler, Nilufar Mossaheb, Gregor Berger, Monika Schlögelhofer, Merete Nordentoft, Eric Y H Chen, Swapna Verma, Dorien H Nieman, Scott W Woods, Barbara A Cornblatt, William S Stone, Daniel H Mathalon, Carrie E Bearden, Kristin S Cadenhead, Jean Addington, Elaine F Walker, Tyrone D Cannon, Mary Cannon, Pat McGorry, Paul Amminger, Gerard Cagney, Barnaby Nelson, Clark Jeffries, Diana Perkins, David R Cotter

**Affiliations:** Department of Psychiatry, Royal College of Surgeons in Ireland, University of Medicine and Health Sciences, Dublin, Ireland; SFI FutureNeuro Research Centre, Royal College of Surgeons in Ireland, University of Medicine and Health Sciences, Dublin, Ireland; Department of Psychiatry, Royal College of Surgeons in Ireland, University of Medicine and Health Sciences, Dublin, Ireland; Department of Psychology, Royal College of Surgeons in Ireland, Dublin 2, Ireland; Department of Psychiatry, Royal College of Surgeons in Ireland, University of Medicine and Health Sciences, Dublin, Ireland; Department of Psychiatry, Royal College of Surgeons in Ireland, University of Medicine and Health Sciences, Dublin, Ireland; Department of Psychiatry, Royal College of Surgeons in Ireland, University of Medicine and Health Sciences, Dublin, Ireland; Centre for Public Health, Queen’s University Belfast, Belfast, UK; School of Biomolecular and Biomedical Science, Conway Institute, University College Dublin, Dublin, Ireland; School of Pharmacy and Pharmaceutical Sciences, Cardiff University, Wales, UK; School of Pharmacy and Pharmaceutical Sciences, Cardiff University, Wales, UK; Department of Psychiatry, Academic Medical Center, Amsterdam, The Netherlands; Brain and Mind Centre, The University of Sydney, Sydney, NSW, Australia; Department of Psychiatry, Jena University Hospital, Jena, Germany; Centre for Youth Mental Health, University of Melbourne, Parkville, VIC, Australia; Orygen, The National Centre of Excellence in Youth Mental Health, Melbourne, VIC, Australia; Centre for Youth Mental Health, University of Melbourne, Parkville, VIC, Australia; Orygen, The National Centre of Excellence in Youth Mental Health, Melbourne, VIC, Australia; Centre for Youth Mental Health, University of Melbourne, Parkville, VIC, Australia; Institute for Mental and Physical Health and Clinical Translation (IMPACT), Deakin University, Geelong, VIC, Australia; School of Health Sciences, University of Manchester, Manchester, UK; Centre for Youth Mental Health, University of Melbourne, Parkville, VIC, Australia; Orygen, The National Centre of Excellence in Youth Mental Health, Melbourne, VIC, Australia; Medical Faculty, University of Basel, Switzerland; Department of Psychiatry and Psychotherapy, Clinical Division of Social Psychiatry, Medical University of Vienna, Vienna, Austria; Department of Child and Adolescent Psychiatry and Psychotherapy, Psychiatric Hospital, University of Zurich, Zurich, Switzerland; BioPsyC—Biopsychosocial Corporation, Non-profit Association for Research Funding Ltd, Vienna, Austria; Mental Health Center Copenhagen, Research Unit (CORE), Copenhagen, Denmark; Department of Clinical Medicine, Faculty of Health and Medical Sciences, University of Copenhagen, Copenhagen, Denmark; Department of Psychiatry, School of Clinical Medicine, LKS Faculty of Medicine, The University of Hong Kong, 2/F New Clinical Building, Queen Mary Hospital, Pok Fu Lam, Hong Kong; The State Key Laboratory of Brain and Cognitive Sciences, The University of Hong Kong, Pok Fu Lam, Hong Kong; Office of Education, Duke-NUS Graduate Medical School, Singapore, Singapore; Department of Psychosis & East Region, Institute of Mental Health, Singapore, Singapore; Department of Psychiatry, Academic Medical Center, Amsterdam, The Netherlands; Department of Psychiatry, Yale University School of Medicine, New Haven, CT, USA; Department of Psychiatry, Zucker Hillside Hospital, Long Island, NY, USA; Department of Psychiatry, Harvard Medical School at Beth Israel Deaconess Medical Center and Massachusetts General Hospital, Boston, MA, USA; Department of Psychiatry and Behavioral Sciences, University of California, San Francisco, San Francisco, CA, USA; Mental Health Service 116d, Veterans Affairs San Francisco Health Care System, San Francisco, CA, USA; Semel Institute for Neuroscience and Human Behavior, Departments of Psychiatry and Biobehavioral Sciences and Psychology, University of California, Los Angeles, CA, USA; Department of Psychiatry, University of California, San Diego, CA, USA; Department of Psychiatry, Hotchkiss Brain Institute, University of Calgary, Calgary, Canada; Department of Psychology, Emory University, Atlanta, GA, USA; Department of Psychiatry, Emory University, Atlanta, GA, USA; Department of Psychology, Yale University, New Haven, CT, USA; Department of Psychiatry, Yale University, New Haven, CT, USA; Department of Psychiatry, Royal College of Surgeons in Ireland, University of Medicine and Health Sciences, Dublin, Ireland; SFI FutureNeuro Research Centre, Royal College of Surgeons in Ireland, University of Medicine and Health Sciences, Dublin, Ireland; Department of Psychiatry, Beaumont Hospital, Dublin 9, Ireland; Centre for Youth Mental Health, University of Melbourne, Parkville, VIC, Australia; Orygen, The National Centre of Excellence in Youth Mental Health, Melbourne, VIC, Australia; Centre for Youth Mental Health, University of Melbourne, Parkville, VIC, Australia; Orygen, The National Centre of Excellence in Youth Mental Health, Melbourne, VIC, Australia; School of Biomolecular and Biomedical Science, Conway Institute, University College Dublin, Dublin, Ireland; Centre for Youth Mental Health, University of Melbourne, Parkville, VIC, Australia; Orygen, The National Centre of Excellence in Youth Mental Health, Melbourne, VIC, Australia; Renaissance Computing Institute, University of North Carolina, Chapel Hill, NC, USA; Department of Psychiatry, University of North Carolina, Chapel Hill, NC, USA; Department of Psychiatry, Royal College of Surgeons in Ireland, University of Medicine and Health Sciences, Dublin, Ireland; SFI FutureNeuro Research Centre, Royal College of Surgeons in Ireland, University of Medicine and Health Sciences, Dublin, Ireland; Department of Psychiatry, Beaumont Hospital, Dublin 9, Ireland

**Keywords:** psychosis, prediction, proteome, complement, coagulation, model, high risk, immune

## Abstract

Psychosis risk prediction is one of the leading challenges in psychiatry. Previous investigations have suggested that plasma proteomic data may be useful in accurately predicting transition to psychosis in individuals at clinical high risk (CHR). We hypothesized that an a priori-specified proteomic prediction model would have strong predictive accuracy for psychosis risk and aimed to replicate longitudinal associations between plasma proteins and transition to psychosis. This study used plasma samples from participants in 3 CHR cohorts: the North American Prodrome Longitudinal Studies 2 and 3, and the NEURAPRO randomized control trial (total *n* = 754). Plasma proteomic data were quantified using mass spectrometry. The primary outcome was transition to psychosis over the study follow-up period. Logistic regression models were internally validated, and optimism-corrected performance metrics derived with a bootstrap procedure. In the overall sample of CHR participants (age: 18.5, *SD*: 3.9; 51.9% male), 20.4% (*n* = 154) developed psychosis within 4.4 years. The a priori-specified model showed poor risk-prediction accuracy for the development of psychosis (*C*-statistic: 0.51 [95% CI: 0.50, 0.59], calibration slope: 0.45). At a group level, Complement C8B, C4B, C5, and leucine-rich α-2 glycoprotein 1 (LRG1) were associated with transition to psychosis but did not surpass correction for multiple comparisons. This study did not confirm the findings from a previous proteomic prediction model of transition from CHR to psychosis. Certain complement proteins may be weakly associated with transition at a group level. Previous findings, derived from small samples, should be interpreted with caution.

## Introduction

There is mounting evidence for the effectiveness of early intervention in psychosis.^1,2^ Psychosis risk-enriched populations have been identified, using prodromal symptom-based approaches (eg, individuals at clinical high risk [CHR])^3^ and systems-based approaches (eg, child and adolescent mental health service use or hospital presentation for self-harm^4,5^). Several studies have attempted to predict psychosis at an individual level in CHR populations,^6^ aiming to further improve detection and early intervention approaches. Other studies have highlighted poor functioning among individuals with CHR symptoms regardless of transition to psychosis^7^ and developed prediction models for future functioning.^8–10^

Accumulating evidence suggests that dysregulation in peripheral proteins is evident prior to the onset of psychosis.^11–13^ Specifically, it has been proposed that changes in the complement and coagulation pathways, which are known to be involved in defense against pathogen infection and injury, may confer vulnerability to psychosis.^14^ Levels of proteins involved in these pathways have also been shown to associate with outcomes after a first episode of psychosis.^15^ Alpha-2-macroglobulin (A2M; a broad-spectrum proteinase inhibitor of thrombin, Factor Xa, and plasmin which is structurally related to complement components 3 and 4^16,17^) has been reported as being differentially expressed in the blood of individuals across the psychosis spectrum.^11,13,18–20^ In a previous study,^13^ A2M in particular was identified as a promising predictor of transition to psychosis.

A recent systematic review^21^ found that while several different prognostic models have been developed to predict psychosis among individuals with CHR symptoms using proteomic, lipidomic, or genetic data,^13,19,22–24^ there has been limited replication of findings or external validation of models, which has been recognized as an important limitation in the field.^21,25,26^ Using a large multi-study population, we aimed to clarify the potential of plasma proteins, quantified using proteomic methods, to predict transition to psychosis among individuals at CHR. We hypothesized that an a priori*-*specified prediction model would have a strong predictive ability for psychosis risk. As secondary aims, we investigated exploratory proteomic models of psychosis risk and longitudinal associations of individual proteins with transition to psychosis. Furthermore, we developed proteomic prediction models and investigated longitudinal associations with functioning as a secondary outcome.

## Methods

This study is reported according to the TRIPOD guidelines for transparent reporting of studies on prediction models for individual prognosis or diagnosis.^27^

### Participants

Participants involved in this investigation were part of 3 multisite studies, the North American Prodrome Longitudinal Study (NAPLS) 2,^28^ a later wave of the same study with a new, independent cohort; NAPLS3^29^ and the NEURAPRO study; a multicenter randomized controlled trial of omega-3 polyunsaturated fatty acids vs placebo in young people at ultra-high risk of psychotic disorders.^30^ Together, 754 individuals at high risk of psychosis who provided plasma samples at baseline and had follow-up outcome data available were included in this investigation. A brief description of each sample is provided below.

#### NAPLS2 and NAPLS3.

NAPLS2 and NAPLS3 are multisite studies from North America and include prospective cohort data on individuals at CHR for psychosis across 8 and 9 sites, respectively.^28,29^ NAPLS2 participants were recruited between 2008 and 2013. NAPLS3 participants were recruited between 2015 and 2018. Individuals with CHR symptoms were referred from health care providers, educators, or social service agencies or self-referred as a result of community outreach.^28^ Individuals, aged between 12 and 30 years, were screened for suitability and then assessed with the Structured Interview for Psychosis-risk Syndromes (SIPS)^31,32^ to determine if they met Criteria of Psychosis-Risk Syndromes. Baseline and follow-up (6-, 12-, 18-, and 24-month) interviews were conducted to assess various clinical outcomes, including transition to psychosis. CHR participants who provided a blood sample and either transitioned to psychosis or did not transition to psychosis and were followed for a minimum of 2 years were included in this investigation (NAPLS2 *n* = 222, NAPLS3 *n* = 261). Characteristics of participants who did and did not provide a blood sample are detailed in supplementary table 1. Blood samples used for the purpose of this investigation were drawn at baseline into EDTA plasma tubes. Processing time varied with an interquartile range of 40–79 min. Samples were stored in aliquots at −80°C. NAPLS2 and NAPLS3 samples underwent 2 and 1 freeze-thaw cycles, respectively, prior to analysis.

#### NEURAPRO.

The NEURAPRO study was an international multisite randomized, double-blind, placebo-controlled trial (ACTRN: 12608000475347) examining the efficacy of omega-3 polyunsaturated fatty acids to prevent transition to psychosis in participants at CHR of psychosis.^30^ The NEURAPRO trial ran in 10 international sites across Australia, Austria, Germany, Denmark, Netherlands, Switzerland, China, and Singapore in established early psychosis centers, from 2010 to 2014. Participants, aged 13–40 who were referred to these treatment centers, were approached for participation in the trial if they met at-risk criteria (herein referred to as CHR). CHR criteria were assessed using the Comprehensive Assessment of At-Risk Mental State (CAARMS).^30,33^ Follow-up interviews were conducted at 6, 12, and 24 months. CHR participants who provided a blood sample were included in this investigation (*n* = 271). Blood samples used for the purpose of this investigation were drawn at baseline. Blood samples were drawn into EDTA plasma tubes and processed within 90 min. Samples were stored in aliquots at −80°C and underwent 2 freeze-thaw cycles prior to analysis.

### Mass Spectrometry and Bioinformatics

Detailed description of the sample preparation and mass spectrometry (MS) methods used in this investigation are described previously.^34^ Sample processing and MS analysis were performed blind to transition status, after the outcomes were determined in both studies. Briefly, samples were randomized following a block randomization design^35^ (prepared by a separate researcher) that preserved the transition rate and the proportion of samples from each study across the randomization sequence. Plasma samples were prepared for MS using PreOmics kits (PreOmics GmbH, Munich, Germany). Samples were then transferred to Evosep tips (Evosep, Odense, Denmark) and eluted. Samples were analyzed in a Bruker timsTof Pro mass spectrometer (Bruker, Massachusetts, United States) connected to an Evosep One liquid chromatography system that injected the samples. Internal standards were included at regular intervals between samples. Further details can be found in Supplementary Methods.

MaxQuant^36^ was used to analyze the raw MS files and derive label-free quantification (LFQ) values. Proteins that were quantified in more than 70% of samples were brought forward for analysis.

### Outcomes

The primary outcome was transition to psychosis. For NAPLS2/NAPLS3, the median time from baseline to psychosis transition was 7.5 months, ranging from 1 week to 4.4 years. For NEURAPRO, the median time from baseline to psychosis transition was 7 months, ranging from 2.5 weeks to 4.3 years. The secondary outcome was functioning at 24 months follow-up as measured in each of the studies (Global Assessment of Functioning in NAPLS2/3; Social and Occupational Functioning Assessment Scale in NEURAPRO). These scales are not directly comparable as the Global Assessment of Functioning scale incorporates symptom severity, while the Social and Occupational Functioning Assessment Scale measures social and occupational functioning independent of symptoms. Further details can be found in Supplementary Materials.

### Ethics

Ethics committee approval was obtained for the NEURAPRO, NAPLS2, and NAPLS3 studies at each individual site.^28–30^ Ethics approval for the plasma biomarker analysis in this study was obtained from the Royal College of Surgeons in Ireland Research Ethics Committee (REC No. 202211009).

### Data analysis

#### Missing Protein Data and Pre-processing.

LFQ values for each protein were log_2_ transformed. Missing LFQ values were treated as left-censored missing data (missing not-at-random; assumed to be below the limit of detection). Using a left-censored imputation approach, missing values were replaced with values drawn from a normal distribution centered at the first percentile of a protein’s overall distribution. The normal distribution centered at the first percentile had its own variation in a ratio of 0.5 times the standard deviation of values for a protein. Values for each protein were subsequently standardized and winsorized at 4 standard deviations above and below the mean.

#### Model Predictor Selection.

Using *pmsampsize* in *R*,^37^ it was estimated that given the overall sample size, including no more than 11 predictors in the models with binary outcomes would minimize overfitting. Two a priori-specified models were developed: a model predicting transition in the overall sample and a model predicting Global Assessment of Functioning in NAPLS2/NAPLS3. Ten predictors were prespecified for inclusion in the models based on results from a previous study^13^: A2M, Ig mu chain C region (IGHM), Complement component C6 (C6), Clusterin (CLU), Plasminogen (PLG), Carboxypeptidase N subunit 2 (CPN2), Vitamin K-dependent protein S (PROS1), Vitamin D-binding protein (GC), Complement C1s subcomponent (C1S), and Transthyretin (TTR). These proteins were chosen by considering their contribution to the original and replication analyses in Mongan et al.^13^ and how reliably the proteins were measured in the current study (assessed by their coefficient of variation [CV] and their percentage missing values prior to imputation; see supplementary table 2). For the model predicting follow-up Global Assessment of Functioning score, the 10 proteins were included as predictors as well as an additional predictor, baseline Global Assessment of Functioning score. For the exploratory model predicting transition, all 99 proteins quantified by MS were included as predictors in the model. Continuous predictors were modeled as linear, and no interaction terms were included in the models.

#### Model Development and Internal Validation.

For the a priori*-*specified models, logistic, and linear regression models with bootstrapped shrinkage of coefficients were implemented for outcomes of transition and Global Assessment of Functioning, respectively. A logistic regression model with elastic net penalization was implemented for the exploratory prediction of transition.

The primary performance metrics for models with a binary outcome were the *C*-statistic and the calibration slope. The performance of the models predicting transition were examined in the overall sample. Given that this study incorporates several separate samples, additional sample-stratified investigations were conducted. The primary performance metrics for models with continuous outcomes were *R*^2^ (proportion of variance in the dependent variable explained) and mean squared error.

The a priori*-*specified models were internally validated by using 1000 bootstrap resamples to derive optimism-corrected performance metrics.^38^ A nested cross-validation procedure was used for the exploratory model development and internal validation. There were 8 outer cross-validation folds, derived from study sites, which were used for model evaluation. Further details are found in Supplementary Methods.

#### Proteomic Associations.

We examined the differential expression of CHR samples from individuals who transitioned to psychosis from those who did not, using logistic regression adjusting for age, sex, and study. This was conducted on the overall sample and within each cohort separately. We examined associations between protein levels and functioning at 24 months follow-up, as measured in each of the studies, using linear regression adjusting for age and sex. Additional cohort-stratified analyses were carried out, further adjusting for body mass index (BMI) where available (in NAPLS3 and NEUARPRO). Corrections for multiple comparisons were applied to associations with binary and continuous outcomes using the Benjamini-Hochberg method with a 5% false discovery rate (FDR).

#### Enzyme-linked Immunosorbent Assay (ELISA) Validation.

Our previous investigation indicated that A2M was differentially expressed at baseline between those who transitioned to psychosis and those who did not and had the largest single contribution to the prediction of transition.^13^ Using the results from the previous investigation we calculated the sample size required to replicate these findings in the current study with 90% power. Thus, for a subsample of participants (*n* = 142), we measured plasma A2M concentrations using ELISA (a separate protein measurement technique) and investigated the association of ELISA A2M levels with transition. Further details on the ELISA procedure can be found in Supplementary Methods.

In a subsample of NAPLS2 participants (*n* = 81), multiplex immunoassay data (Myriad Rules-Based Medicine, Human Discovery Map assay) were available from a previous study.^23^ To validate the MS data, we calculated the Spearman’s rank correlation between A2M measured with MS and with the Human Discovery Map assay.

## Results

### Participant Characteristics

Participant characteristics are detailed in tables 1–3. The overall psychosis transition percentage in this investigation was 20.4% (*n* = 154). Differences in participant characteristics by study are detailed in supplementary results.

**Table 1.** Overall and Cohort-Specific Participant Characteristics Shared Between Included Studies

		Full Sample (*n* = 754)	NEURAPRO (*n* = 271)	NAPLS2 (*n* = 222)	NAPLS3 (*n* = 261)
Age ($\bar{X}$*SD*)	Overall	18.5 (3.9)	19.0 (4.4)	17.9 (3.3)	18.6 (3.8)
	CHR-NT	18.5 (3.9)	18.9 (4.3)	17.8 (3.2)	18.5 (3.7)
	CHR-T	18.7 (4.2)	19.5 (5.3)	18.1 (3.6)	19.0 (4.0)
Sex (% male)	Overall	51.9 (*n* = 391)	43.9 (*n* = 119)	57.7 (*n* = 128)	55.2 (*n* = 144)
	CHR-NT	51.3 (*n* = 308)	44.1 (*n* = 104)	57.1 (*n* = 89)	55.3 (*n* = 115)
	CHR-T	53.9 (*n* = 83)	42.9 (*n* = 15)	59.1 (*n* = 39)	54.7 (*n* = 29)
BMI ($\bar{X}$*SD*)	Overall	24.2 (5.7)	23.9 (5.4)	—	24.4 (6.0)
	CHR-NT	24.2 (5.7)	23.9 (5.2)	—	24.6 (6.1)
	CHR-T	24.0 (6.0)	24.1 (6.9)	—	24.0 (5.4)

*Note*: BMI, body mass index; CHR-NT, clinical high risk non-transition; CHR-T, clinical high risk transition.

**Table 2.** Cohort-Specific Participant Characteristics: NAPLS2 and NAPLS3

NAPLS2 and NAPLS 3 (*n* = 483)				
		Overall	CHR-NT	CHR-T
Ethnicity (%) (*n* = 483)	European	55.1 (*n* = 266)	56.0 (*n* = 204)	52.1 (*n* = 62)
	Interracial	14.5 (*n* = 70)	13.5 (*n* = 49)	17.6 (*n* = 21)
	African	13.9 (*n* = 67)	13.7 (*n* = 50)	14.3 (*n* = 17)
	Central or South American	4.8 (*n* = 23)	4.7 (*n* = 17)	5.0 (*n* = 6)
	East Asian	4.8 (*n* = 23)	5.8 (*n* = 21)	1.7 (*n* = 2)
	South Asian	2.7 (*n* = 13)	2.5 (*n* = 9)	3.4 (*n* = 4)
	Southeast Asian	1.9 (*n* = 9)	1.6 (*n* = 6)	2.5 (*n* = 3)
	First Nations	1.9 (*n* = 9)	2.2 (*n* = 8)	0.8 (*n* = 1)
	Other	0.6 (*n* = 3)	0 (*n* = 0)	2.5 (*n* = 3)
Tobacco use (%) (*n* = 479)	None	78.5 (*n* = 379)	80.1 (*n* = 290)	76.1 (*n* = 89)
	Occasionally	10.0 (*n* = 48)	9.7 (*n* = 35)	11.1 (*n* = 13)
	<10 times a day	6.0 (*n* = 29)	5.5 (*n* = 20)	7.7 (*n* = 9)
	>10 times a day	4.8 (*n* = 23)	4.7 (*n* = 17)	5.1 (*n* = 6)
Baseline GAF ($\bar{X}$*SD*) (*n* = 481)		50.0 (11.5)	50.9 (11.4)	47.1 (11.3)
Baseline SOPS Positive ($\bar{X}$*SD*) (*n* = 483)		12.5 (3.2)	12.1 (3.5)	13.8 (3.6)
Baseline SOPS Negative ($\bar{X}$*SD*) (*n* = 478)		12.0 (6.1)	11.8 (5.9)	12.8 (6.5)
Medication use (%) (*n* = 482)	Antipsychotics	19.7 (*n* = 95)	17.1 (*n* = 62)	27.7 (*n* = 33)
	Antidepressants	30.9 (*n* = 149)	30.6 (*n* = 111)	31.9 (*n* = 38)

*Note*: GAF, global assessment of functioning; SOPS, scale of prodromal symptoms.

**Table 3.** Cohort-Specific Participant Characteristics: NEURAPRO

NEURAPRO (*n* = 271)				
		Overall	CHR-NT	CHR-T
Ethnicity (%) (*n* = 267)	Caucasian	80.9 (*n* = 216)	81.9 (*n* = 190)	74.3 (*n* = 26)
	Asian	13.1 (*n* = 35)	12.9 (*n* = 30)	14.3 (*n* = 5)
	Other	6.0 (*n* = 16)	5.2 (*n* = 12)	11.4 (*n* = 4)
Tobacco use in last year (%) (*n* = 265)	Never	44.2 (*n* = 117)	44.3 (*n* = 102)	42.9 (*n* = 15)
	Once or twice	6.0 (*n* = 16)	7.0 (*n* = 16)	0 (*n* = 0)
	Monthly	3.4 (*n* = 9)	2.6 (*n* = 6)	8.6 (*n* = 3)
	Weekly	7.5 (*n* = 20)	7.4 (*n* = 17)	8.6 (*n* = 3)
	Daily	38.9 (*n* = 103)	38.7 (*n* = 89)	40.0 (*n* = 14)
Baseline SOFAS (*SD*) (*n* = 265)		53.9 (12.2)	54.2 (12.6)	51.4 (9.2)
Baseline CAARMS Positive ($\bar{X}$*SD*) (*n* = 271)		37.1 (16.9)	36.7 (17.0)	39.7 (16.3)
Baseline SANS Composite ($\bar{X}$*SD*) (*n* = 265)		18.0 (13.0)	17.3 (13.1)	23.0 (11.9)

*Note:* CAARMS, comprehensive assessment of at-risk mental state; SANS, scale for the assessment of negative symptoms; SOFAS, social and occupational functioning assessment scale.

There were no significant differences between participants who developed psychosis and those who did not in terms of age (*t*(226) = −0.69, *P* = .49), sex (*χ*^2^(1) = 0.32, *P* = .57) or BMI (*t*(117) = 0.30, *P* = .77).

### MS

We identified 99 proteins that were quantified in more than 70% of samples. The 10 a priori selected proteins (A2M, IGHM, C6, CLU, PLG, CPN2, PROS1, GC, C1S, and TTR) were measured with a mean CV of 13.5% (range: 6.8% [A2M] to 21.3% [C6]) and had mean of 0.4% missing values (range: 0.0% [A2M] to 2.0% [C6]). The CV and percentage missingness for each protein are presented in supplementary table 2.

### Prediction Modeling

#### A Priori-specified Model Predicting Transition.

The a priori*-*specified proteomic model of transition had an optimism-corrected *C*-statistic of 0.51 (95% CI: 0.50, 0.59) and an optimism-corrected calibration slope of 0.45. A calibration plot for the a priori-specified model is presented in Supplementary figure 1. Cohort-stratified analyses revealed similar results (NAPLS2 *C*-statistic: 0.56 [95% CI: 0.53, 0.69], calibration slope 0.51; NAPLS3 *C*-statistic: 0.54 [95% CI: 0.52, 0.66], calibration slope 0.47; NEURAPRO *C*-statistic: 0.49 [95% CI: 0.48, 0.65], calibration slope 0.32). These results indicated that the a priori proteomic model had a poor ability to predict transition to psychosis.

#### Exploratory Model Predicting Transition.

The exploratory model developed to predict transition included all 99 proteomic predictors available and had a mean cross-validated *C*-statistic of 0.46. Cohort-stratified results were similar (NAPLS2 *C*-statistic: 0.56; NAPLS3 *C*-statistic: 0.46; and NEURAPRO *C*-statistic: 0.53).

#### A Priori-specified Model Predicting Global Assessment of Functioning.

An a priori*-*specified model was developed with data from NAPLS2 and NAPLS3 participants with Global Assessment of Functioning score at 24 months follow-up as a continuous outcome. The model included the 10 a priori selected proteins and baseline Global Assessment Functioning score as predictors. The a priori*-*specified model had an optimism-corrected *R*^2^ of 0.058 and a mean squared error of 176.4. In comparison, a model developed with Global Assessment of Functioning as the only predictor had an optimism-corrected *R*^2^ of 0.081 and a mean squared error of 173.4.

### Proteomic Associations

#### Overall.

Of the 99 proteins within this dataset, Complement component C8 beta chain (C8B), Complement C4-B (C4B), Leucine-rich α-2 glycoprotein (LRG1), and Complement C5 (C5) were differentially expressed between those who transitioned and those who did not transition to psychosis while adjusting for age, sex, and study. C8B levels were positively associated with transition, while C4B, C5, and LRG1 were inversely associated with transition. However, these associations did not surpass FDR correction for multiple comparisons (table 4; supplementary table 3). A2M had an odds ratio of 1.23 for transition to psychosis (95% CI: 1.00, 1.52; *P* = 0.052).

**Table 4.** The Top 10 Proteins Associated with Transition to Psychosis in the Overall Sample

Protein	Odds	LCI	UCI	*P*-Value	FDR *P*-Value
C8B	1.258	1.039	1.523	.019	.664
C4B	0.809	0.677	0.968	.021	.664
LRG1	0.810	0.675	0.974	.025	.664
C5	0.825	0.685	0.994	.043	.664
C4A	0.835	0.697	1.000	.051	.664
A2M	1.231	0.998	1.519	.052	.664
FGG	1.200	0.998	1.442	.052	.664
APOL1	0.844	0.707	1.007	.060	.664
FGB	1.192	0.989	1.437	.065	.664
CFB	0.845	0.705	1.014	.071	.664

*Note*: Analyses are adjusted for age and sex. FDR, false discovery rate; LCI, lower confidence interval; UCI, upper confidence interval.

#### NAPLS2 and NAPLS3.

C4B, Complement C4-A (C4A), Complement C2 (C2), A2M, LRG1, and the Fibrinogen alpha, beta, and gamma chains (FGA, FGB, and FGG) were differentially expressed between those who transitioned to psychosis and those who did not, adjusting for age, sex, and study. However, these associations did not surpass FDR Correction (supplementary table 4). In analyses additionally adjusting for antipsychotic use, results were similar; LRG1 was no longer associated with transition, while Vitronectin (VTN) was associated with transition prior to FDR correction (supplementary table 5). In analyses additionally adjusting for BMI (available in NAPLS3 only), only C6 was associated with transition, but the association did not surpass correction for multiple comparisons (supplementary table 6).

Global Assessment of Functioning score at 24 months follow-up was investigated as a continuous outcome in NAPLS2 and NAPLS3. Complement C1r subcomponent (C1R), Lumican (LUM), Retinol-binding protein 4 (RBP4), C1S, C6, and Plasma protease C1 inhibitor (SERPING1) were associated with follow-up Global Assessment of Functioning score adjusting for age and sex (table 5, supplementary table 7). These associations did not surpass correction for multiple comparisons.

**Table 5.** The Top 10 Proteins Associated with Global Assessment of Functioning at 24 Months Follow-up in NAPLS2 and NAPLS3

Protein	Beta Coefficient	LCI	UCI	*P*-Value	FDR *P*-Value
C1R	−1.784	−3.159	−0.408	.011	.741
LUM	−1.741	−3.218	−0.263	.021	.741
RBP4	1.55	0.145	2.954	.031	.741
C1S	−1.482	−2.858	−0.107	.035	.741
C6	−1.447	2.821	−0.074	.039	.741
SERPING1	−1.442	−2.85	−0.033	.045	.741
TTN	−1.239	−2.575	0.098	.069	.979
F2	1.221	−0.177	2.618	.087	.996
APOH	1.114	−0.24	2.469	.106	.996
IGKV1.8.9	1.075	−0.301	2.451	.125	.996

*Note*: Analyses are adjusted for age and sex. FDR, false discovery rate; LCI, lower confidence interval; UCI, upper confidence interval.

#### NEURAPRO.

C5 was differentially expressed between those who transitioned to psychosis and those who did not while adjusting for age, sex, and BMI. This association did not surpass FDR Correction (supplementary table 8).

The Social and Occupational Functioning Scale score at 24 months follow-up was investigated as a continuous outcome in NEURAPRO. There were no proteins associated with follow-up Social and Occupational Functioning adjusting for age, sex, and BMI (supplementary table 9).

### ELISA Validation of A2M

We compared the A2M ELISA levels of 71 transition and 71 non-transition samples (mean [*SD*]: 5.82 µg/ml [2.07], 6.67 µg/ml [2.46], respectively). In logistic regression analyses adjusting for age, sex, and sample storage time, A2M concentrations were not associated with transition status (OR: 0.91; 95% CI: 0.77, 1.07; *P*-value = .262). The MS measurements and multiplex immunoassay measurements for A2M had a Spearman’s rho correlation of 0.50 (supplementary table 10).

## Discussion

Combining data from 3 international studies spanning America, Europe, Asia, and Australia, in the largest investigation of this research question to date, we find no support for individual level prediction of transition to psychosis in CHR patients using proteomic data. Neither our a priori nor exploratory analyses replicated the accurate proteomic prediction of psychosis seen in previous studies, particularly from Mongan et al.^13^. However, in line with previous studies, we observed weak longitudinal associations between complement and coagulation-related proteins and transition to psychosis.

Currently, there is a lack of replication, validation, or implementation of prediction models in medicine.^6,39^ We followed the latest guidance in developing the models in this study.^40,41^ This investigation ensured that there was a large enough sample size to fit 10 predictors in a model, based on recommended sample size calculations.^37^ Samples were block-randomized before proteomic measurement. We chose model predictors blind to their association with transition status in our sample, based on their previously reported performance as predictors of transition to psychosis.^13^ Furthermore, only predictors with little or no missing data that were measured reliably were included. Finally, we internally validated the performance of our models through bootstrapping or nested cross-validation. The most plausible reasons for the discrepancy between the performance of our prediction model and the performance of models from previous studies^13,19,23^ may be the differences in sample sizes and the different protein measurement techniques employed.

Although the longitudinal associations between proteins and transition status in this study did not surpass corrections for multiple comparisons, the results are in support of previous studies that suggested a longitudinal relationship between complement and coagulation dysregulation and psychosis.^11–13,18,19^ However, the directions of effect are not consistent across studies, including this study. In the study preceding this,^13^ lower A2M levels were associated with transition to psychosis. We now observe a weak but positive relationship between A2M levels and psychosis risk, in line with previously reported differences between individuals with schizophrenia and controls^20^ and a prediction model developed in a schizophrenia case-control sample.^19^ In agreement with a recent study measuring complement proteins with ELISA in individuals with CHR symptoms,^42^ we observe decreased levels of C5 in individuals that transitioned to psychosis.

It may be that dysregulation of complement and coagulation is a risk factor for psychosis in a broad sense, such that even if different aspects of the pathways are dysregulated, the result is still an increased risk of psychosis. If complement or coagulation dysregulation represents a vulnerability to infection or is reflective of a past or prenatal infection, as has previously been suggested,^14^ this could be an explanation for how different aspects of the complement or coagulation pathways can be dysregulated in different individuals, but still lead to an enhanced risk of psychosis.^43–47^ On the other hand, individuals who transition to psychosis are a heterogeneous group who are exposed to a wide variety of risk factors.^48^ For some individuals, other risk factors for psychosis will play a stronger role, and complement or coagulation pathway dysregulation may be most relevant only for a subgroup of individuals who transition to psychosis.

The results in relation to the secondary outcome of functioning at 24 months are similar to those of previous studies. In a previous analysis of the NEURAPRO study, a model developed using the plasma proteome was a poor predictor of future functioning among individuals with CHR symptoms,^34^ in line with the poor performance of the proteomic model predicting functioning among NAPLS participants in this study. The longitudinal associations between proteins and future functioning among NAPLS participants again suggested the involvement of complement system proteins (C1R, C1S, C6, SERPING1 [an inhibitor of C1R]). In a first-episode psychosis cohort, C1R was also inversely associated with follow-up functioning,^15^ which highlights the potential relevance of this complement protein to a crucial clinical outcome across different clinical stages of psychosis.

There are several other explanations for the discrepancy between the results of this study and the previous study which we attempted to replicate.^13^ The sample sizes between the studies differ substantially; there were 49 events (transitions to psychosis) included in the analysis by Mongan et al., while there were 154 events included in the present analysis. The average age of the participants in this study was 19, compared with 23 in the previous study, and the present study had greater ethnic diversity. In the NAPLS studies, there were higher rates of antipsychotic use than in the study by Mongan et al., however, our sensitivity analyses additionally adjusting for antipsychotic use were in agreement with our main analyses of associations with transition.

The effect of BMI on the results should also be considered. In sensitivity analyses additionally adjusting for BMI (where available), C6 was nominally associated with transition in NAPLS3, while C5 was nominally associated with transition in NEURAPRO. The degree to which the associations between complement proteins and transition to psychosis are confounded by BMI are unclear, as the sensitivity analyses had reduced sample sizes and, therefore, reduced power compared with the main analysis.

It should be acknowledged that the NEURAPRO study is a randomized controlled trial, and while there was no main effect observed of the studied intervention (omega-3 fatty acids) on transition to psychosis, the intervention may still have altered levels of plasma proteins. Furthermore, the trial was subject to more stringent exclusion criteria than participants included in Mongan et al.^13^; participants with “abnormal coagulation profile parameters or thyroid function test results >10% above or below the limits of the normal range” were excluded to reduce the risk of adverse events^30^ which could bias participant protein levels. However, our cohort-stratified results excluding NEURAPRO participants did not differ substantially from our main results.

As is common in longitudinal cohort studies, all the studies included in this investigation are prone to selection and attrition bias. It has previously been highlighted that high-risk paradigms based on prodromal symptoms or a combination of reduced functioning and genetic risk may only capture a fraction of individuals who present with psychotic disorder.^49–52^ The participants included in this investigation may not be representative of all individuals who develop a first episode of psychosis. Other groups at risk of psychosis with an enhanced predictive capacity of first episodes have been recently highlighted, including individuals attending CAMHS,^5^ individuals with thoughts of self-harm,^53^ and individuals presenting to hospital after self-harm.^4^ Future studies could investigate the longitudinal associations between complement and coagulation proteins and psychosis in these or other groups that capture a larger proportion of individuals who develop psychotic disorders. Further promising blood-based markers other than proteins^22,54^ should also be investigated, and may prove most useful for the prediction of psychosis in combination with non-biological risk factors.^55^

This study is limited by the depth of the proteome measured. To accommodate the analysis of a large number of samples with MS, we sacrificed identifying a larger number of proteins, in particular low abundance proteins. However, in line with the aims of this investigation, which was focused on replicating prediction based on certain proteins, the majority of the proteins of interest identified in a previous study^13^ were measured accurately in this current investigation. In this study, proteins in plasma were measured at a single timepoint. Proteins measured in samples from multiple timepoints may provide a more accurate representation of long-standing protein levels, however, using repeated blood measures would also make the implementation of a resulting clinical prediction model more challenging.

In conclusion, the current findings do not support individual-level prediction of transition to psychosis in individuals with CHR symptoms using proteomic data. Due to their inadequate performance, the models developed in this study are not recommended for external validation. In line with previous studies, we observed weak longitudinal associations between complement and coagulation-related proteins and transition to psychosis among a CHR population, suggesting that dysregulation of the complement or coagulation pathways may be weak risk factors for psychosis. In future studies, the potential of proteomic data and other peripheral physiological data for the prediction of transition should be investigated in cohorts capturing a larger proportion of individuals who develop psychotic disorders.

## Supplementary Material

sbad184_suppl_Supplementary_Tables_1-10_Figures_1
