# Supplementary material for: Proteomic Biomarkers for the Prediction of Transition to Psychosis in Individuals at Clinical High Risk: A Multi-cohort Model Development Study
Source: Schizophr Bull. 2024 Jan 19;50(3):579–88. doi: 10.1093/schbul/sbad184 (PMC11059811; doi:10.1093/schbul/sbad184)
Supplement: sbad184_suppl_Supplementary_Tables_1-10_Figures_1 [file sbad184_suppl_supplementary_tables_1-10_figures_1.pdf]

Contents

Supplementary Methods 2

Outcomes 2

Liquid Chromatography with Tandem Mass Spectrometry (Bruker timsTof Pro/Evosep One) 2

Mass Spectrometry Internal Standards 3

Bioinformatics (Maxquant) 3

Model Predictor Budget Calculations 3

Prediction Modelling Details 4

Enzyme-linked Immunoassay (ELISA) 6

Supplementary Results 7

Differences in Participant Characteristics by Study 7

Hi3 E. Coli CV 7

A Priori-Specified Model Equation 7

Exploratory Analyses Stratified by Cohort 7

Supplementary Tables 8

Supplementary Table 1: Characteristics of NAPLS2 and NAPLS3 participants who did and did not provide a blood sample for proteomic analysis. 8

Supplementary Table 2: CV (%) and % missing for each protein. 8

Supplementary Table 3: Associations between proteins and transition status in the overall sample, adjusting for age, sex, and study. 11

Supplementary Table 4: Top ten associations between proteins and transition status in NAPLS2 and NAPLS3, adjusting for age, sex, and study. 13

Supplementary Table 5: Top ten associations between proteins and transition status in NAPLS2 and NAPLS3, adjusting for age, sex, study, and antipsychotic use. 14

Supplementary Table 6: Top ten associations between proteins and transition status in NAPLS3, adjusting for age, sex, study, and BMI. 14

Supplementary Table 7: Associations between proteins and Global Assessment of Functioning at 24 months follow-up in NAPLS2 and NAPLS3. 15

Supplementary Table 8: Top ten associations between proteins and transition status in NEURAPRO, adjusting for age, sex, and BMI. 17

Supplementary Table 9: Top ten associations between proteins and Social and Occupational Functioning at 24 months follow-up in NEURAPRO. 18

Supplementary Table 10: Spearman's rho correlation between proteins measured with mass spectrometry and multiplex immunoassay (Myriad Rules Based Medicine, Human Discovery Map). 18

Supplementary Figures 19

Supplementary Figure 1: Calibration plot for the *a-priori* specified model of transition, comparing observed probabilities and expected (predicted) probabilities. 19

Supplementary Methods

Outcomes

NAPLS2 and NAPLS3

Transition to psychosis was defined as meeting the Presence of Psychotic Syndrome (POPS) criteria [1]; at least one of the five Scale of Prodromal Symptoms positive symptoms reaching a level 6 in intensity, for a frequency of >1 hour per day for 4 days per week during the past month or that symptoms seriously impacted functioning to the level of severely disorganised or that the participant presents a danger to themselves or others. The secondary outcome, functioning, was measured with the Global Assessment of Functioning scale [2] at 24 months follow-up in NAPLS2 and NAPLS3.

NEURAPRO

Transition to psychosis was assessed using the CAARMS. Transition to psychosis was defined as daily full-threshold positive psychotic symptoms lasting longer than one week. Participants were re-contacted for further follow up after >2 years and invited for a face-to-face interview [3]. If participants did not consent to a face-to-face interview, they were asked to participate in a brief telephone assessment. If participants could not be contacted, hospital records were consulted, and public health service contact and diagnoses were recorded [3]. The secondary outcome, functioning, was measured with the Social and Occupational Functioning Assessment Scale[4] at 24 months follow-up in NEURAPRO.

Outcomes in both studies were determined before the measurement of proteomic predictors.

Liquid Chromatography with Tandem Mass Spectrometry (Bruker timsTof Pro/Evosep One)

Samples were run on a Bruker timsTof Pro mass spectrometer connected to a Evosep One liquid chromatography system. Tryptic peptides were resuspended in 0.1% formic acid and each sample was loaded on to an Evosep tip. The Evosep tips were placed in position on the Evosep One, in a 96-tip box. The autosampler is configured to pick up each tip, elute and separate the peptides using a set chromatography method (60 samples a day) [5]. Samples were run in one single run.

The mass spectrometer was operated in positive ion mode with a capillary voltage of 1500 V, dry gas flow of 3 l/min and a dry temperature of 180 °C. All data was acquired with the instrument operating in trapped ion mobility spectrometry (TIMS) mode. Trapped ions were selected for ms/ms using parallel accumulation serial fragmentation (PASEF). A scan range of (100-1700 m/z) was performed at a rate of 5 PASEF MS/MS frames to 1 MS scan with a cycle time of 1.03s [6].

Chromatography Buffers:

Buffer B: 99.9% acetonitrile, 0.1% formic acid.

Buffer A: 99.9% water, 0.1% formic acid.

All solvents are LCMS grade.

Mass Spectrometry Internal Standards

Two types of internal standards were included at regular intervals throughout the mass spectrometry run. The first type of internal standard was a pooled sample comprised of plasma samples from the three studies. This standard was spiked with 60 fmol Hi3 E. Coli (Waters Corporations, Massachusetts, United States). The second type of internal standard was commercial pooled plasma (Sigma Aldrich, Missouri, United States, Product code: P9523).

Bioinformatics (Maxquant)

The raw data was searched against the Homo sapiens subset of the Uniprot Swissprot database (reviewed) using the search engine Maxquant (release Version 2.0.3.0) using specific parameters for trapped ion mobility spectra data dependent acquisition (TIMS DDA). Each peptide used for protein identification met specific Maxquant parameters, i.e., only peptide scores that corresponded to a false discovery rate (FDR) of 0.01 were accepted from the Maxquant database search. The normalised protein intensity of each identified protein was used for label free quantitation (LFQ) [7].

Model Predictor Budget Calculations

We used the package *pmsampsize* in R [8] to estimate how many predictors we could include in our model to minimise overfitting. Given our outcome (transition) prevalence of 20.4%, our sample size (n = 754), a shrinkage factor of 0.9 and an estimated C-statistic of 0.75, the *pmsampsize* calculations indicated we should include no more than 11 parameters to minimise overfitting.

Prediction Modelling Details

A priori-specified model predicting transition

The primary performance metrics for models predicting transition were the Concordance (C)-statistic and the calibration slope [9]. The C-statistic is a measure of discrimination (how well the model differentiates between individuals who do and do not develop the outcome). Calibration refers to the agreement between observed outcomes and risk estimates. The calibration slope has an ideal value of 1, were values less than or greater than 1 indicate predicted risks are too extreme or too conservative, respectively.

Optimism-corrected performance metrics were derived using the bootstrap procedure. Optimism-corrected performance has been previously described[10] as the apparent performance of model developed in original sample minus the optimism, where optimism = average(apparent performance of model developed in bootstrap sample − performance of model developed in bootstrap sample and tested in original sample). Non-parametric 95% confidence intervals were derived by subtracting the calculated optimism from the 2.5th and 97.5th percentiles of the bootstrapped C-statistic distribution [11].

Exploratory model predicting transition

We implemented a nested cross-validation framework using python's "sklearn" for parameter tuning and evaluation of model performance. A pre-processing pipeline was developed to prevent information leakage between the pre-processing steps and the model development.

Sklearn logistic regression specification:

solver='saga', penalty = 'elasticnet'

Pipeline order of operations: imputation, winsorisation, standardisation, logistic regression

Grid search parameter ranges:

l1 ratio: 0.0, 0.1, 0.2, 0.3, 0.4, 0.5, 0.6, 0.7, 0.8, 0.9, 1.0.

'C' regularisation parameter: 10^{-2}, 10^{-1}, 10^{0}, 10^{1}, 10^{2}.

The inner cross-validation loop was used to find the optimal combination of l1 ratio and C parameters in a grid search, giving the highest AUC/C-statistic. The inner training folds were cross-validated using

sklearn's "Stratified Shuffle Split" function with 500 random splits and a test size of 0.2. . The ratios of transition status within each random draw were preserved. Parameters that gave the highest AUC/C-statistic in the inner folds were used to specify the models in the corresponding outer evaluation folds. Sklearn's "Group K Fold" was used for the outer cross-validation by study (3 folds comprised of NAPLS2 participants, 3 folds comprised of NAPLS3 participants, and 2 folds comprised of NEURAPRO participants).

We conducted additional sample specific investigations in NAPLS2, NAPLS3 and NEURAPRO. For exploratory model development in NAPLS2 and NAPLS3 only, outer cross-validation by site (NAPLS2 had 8 sites, NAPLS3 had 9 sites) was implemented. For model development in NEURAPRO only, sites were too small to allow for cross-validation by site. Instead, 9 site-independent folds were derived for cross-validation using Sklearn's "StratifiedKFold", which generated folds stratified by transition status.

Continuous outcome – functioning score

A proteomic prediction model was developed in NAPLS2/NAPLS3 for the secondary outcome, Global Assessment of Functioning score at 24 months, and proteomic associations with follow-up functioning were also determined. There was 34.2% missing outcome data due to non-attendance at the month-24 clinic visit. We addressed the missing data through multiple imputation based on Global Assessment of Functioning scores collected at other visits: month 2, 4, 6, 8, 12, and 18.

To facilitate multiple imputation and internal validation we carried out model development in R using the packages "rms" and "Hmisc". Data were imputed 35 times (in accordance with the percentage missing outcome data) for NAPLS2/3 using the function "aregImpute". Model development was carried out in each imputed dataset, using 1000 bootstrap resamples to derive optimism-corrected performance metrics. Internally validated performance metrics were averaged across each imputed dataset.

Proteomic associations with the secondary outcome in NEURAPRO (Social and Occupational Functioning Scale score at 24 months) and in NAPLS2/3 (Global Assessment of Functioning score at 24 months) were also determined using the "aregImpute" function. In NEURAPRO, there was 44.6% missing outcome data due to non-attendance at the month-24 clinic visit. We addressed the missing data through multiple imputation based on Global Assessment of Functioning scores collected at other visits: month 6 and month 12. Data were imputed 45 times for NEURAPRO.

Enzyme-linked Immunoassay (ELISA)

We calculated the number of transition and non-transition samples needed to detect differential expression of A2M with 90% power and an alpha level of 0.05, based on the mean A2M levels observed in Mongan et al., 2020: transition group mean = 1502 (SD: 711.1); non-transition group mean = 1173 (SD: 459.1). The sample size calculation indicated we would require 142 samples (71 transition samples and 71 non-transition samples). We used predominantly NAPLS3 samples for the ELISA as these samples have the shortest storage time and have BMI (body mass index) data available. 71 non-transition samples were randomly selected from the NAPLS3 study. All 53 NAPLS3 transition samples and 18 randomly selected NAPLS2 transition samples were used to make up a total of 71 transition samples. Selected samples were subsequently randomised prior to carrying out the assay.

We used Abcam's (Cambridge, UK) A2M ELISA kit (ab108888) and carried out the ELISA according to the manufacturer's instructions. Samples were spread across the two plates and internal standards were included, spread diagonally across the plates. The internal standards were used to calculate coefficients of variation.

We analysed the data with logistic regression adjusting for age, sex, and sample storage time.

Supplementary Results

Differences in Participant Characteristics by Study

Individual study transition to psychosis percentages in this investigation were 29.7% (n = 66) for NAPLS2, 20.3% (n = 53) for NAPLS3 and 12.9% (n = 35) for NEURAPRO. There were specific differences between the samples in further characteristics. Participants in NAPLS2 were significantly younger than those in the NEURAPRO study (B = -1.1; CI: -1.8, -0.4; p =.003). There was a greater proportion of females in the NEURAPRO sample (56.1%) in comparison to NAPLS2 (42.3%) and NAPLS3 (44.8%; $\chi^2(2)=10.99$, p =.004). There were no significant differences in Body Mass Index (BMI) between participants in NEURAPRO and NAPLS3. BMI data was not available for NAPLS2 participants. NAPLS2 participants had significantly lower baseline Global Assessment of Functioning scores than NAPLS3 participants (t(474) = -2.41, p = 0.017).

Hi3 E. Coli CV

The Hi3 E. Coli standard included in the pooled plasma from each study had a coefficient of variation of 9.6% across the mass spectrometry run.

Model Equation: A Priori-Specified Model Predicting Transition

The final logistic regression model equation with shrinkage applied to *a priori*-specified predictor β coefficients was as follows:

-1.363375 + (A2M* 0.064153) + (IGHM*0.016992) + (C6*0.037797) + (CLU*0.005518) + (PLG*0.020792) + (CPN2*0.005781) + (PROS1*-0.051502) + (GC*-0.061350) + (C1S*0.029978) + (TTR*0.047239)

Due to the inadequate performance of the model, the model is not recommended for external validation.

Exploratory Analyses Stratified by Cohort

NAPLS2: mean C-Statistic = 0.56 (cross-validation over 8 individual sites).

NAPLS3: mean C-Statistic = 0.46 (cross-validation over 9 individual sites).

NEURAPRO: mean C-Statistic = 0.53 (cross-validation over 9 stratified folds (StratifiedKFold)).

Supplementary Tables

Supplementary Table 1: Characteristics of NAPLS2 and NAPLS3 participants who did and did not provide a blood sample for proteomic analysis.

		Not Included (n=991)	Included (n=483)
**Age (X̄ SD)**		18.4 (4.4)	18.3 (3.6)
**Sex (% Male)**		549 (55.4%)	272 (56.3%)
**Ethnicity**	**European**	563 (56.8%)	266 (55.1%)
	**African**	133 (13.4%)	67 (13.9%)
	**Interracial**	122 (12.3%)	70 (14.5%)
	**Central or South American**	52 (5.2%)	23 (4.8%)
	**East Asian**	38 (3.8%)	23 (4.8%)
	**South Asian**	29 (2.9%)	13 (2.7%)
	**First Nations**	18 (1.8%)	9 (1.9%)
	**Southeast Asian**	17 (1.7%)	9 (1.9%)
	**Other**	19 (0.2%)	3 (0.1%)
**SOPS Positive (X̄ SD)**		12.3 (3.7)	12.5 (3.6)
**SOPS Negative (X̄ SD)**		12.0 (6.3)	12.0 (6.1)
**Global Assessment of Functioning (X̄ SD)**		49.5 (11.4)	49.7 (11.5)

Supplementary Table 2: CV (%) and % missing for each protein.

The ten proteins included in the a priori model are highlighted in bold.

Protein Gene Name	CV (%)	Missing values (%)
**A2M**	6.8	0.0
**IGHM**	9.3	0.0
**C6**	21.3	2.0
**CLU**	10.8	0.0
**PLG**	19.6	0.0
**CPN2**	14.5	0.9
**PROS1**	10.8	0.7
**GC**	9.3	0.0
**C1S**	12.9	0.3
**TTR**	19.9	0.0
A1BG	13.5	0.0
AFM	16.6	0.0
AGT	9.8	0.0
AHSG	14.2	0.0
ALB	7.6	0.0
APCS	6.2	0.0
APOA1	4.5	0.0

APOA2	18.8	0.0
APOA4	7.9	0.0
APOB	4.5	0.0
APOC2	14.7	0.8
APOC3	14.3	1.1
APOD	27.6	0.5
APOE	13.5	0.0
APOH	25.3	6.5
APOL1	11.3	0.3
APOM	34.5	18.7
AZGP1	20.5	0.4
C1QC	16.1	25.5
C1R	22.6	0.3
C2	24.3	21.4
C3	4.7	0.0
C4A	21.7	14.2
C4B	4.9	0.0
C4BPA	61.9	6.9
C5	12.4	0.0
C7	27.8	5.7
C8A	18.5	23.6
C8B	16.0	3.2
C9	21.3	0.0
CFB	6.6	0.0
CFH	10.7	0.0
CFI	11.2	0.1
CP	4.7	0.0
F12	11.4	2.1
F2	17.6	0.1
FGA	5.7	0.3
FGB	7.6	0.0
FGG	6.5	0.1
FN1	10.0	0.1
GSN	11.4	0.0
HBA1	5.9	0.0
HBB	4.5	0.0
HP	5.4	0.0
HPR	8.9	0.0
HPX	9.3	0.0
HRG	16.3	0.0
IGFALS	26.2	14.9
IGHA1	13.2	0.1
IGHD	26.7	16.2
IGHG1	9.9	0.0

IGHG2	16.2	0.0
IGHG3	19.1	0.0
IGHG4	17.9	0.5
IGHV3-30	15.6	13.9
IGHV3-49	19.5	8.5
IGHV5-51	15.5	32.2
IGKC	8.1	0.0
IGKV1-9	27.0	12.7
IGKV2D-28	12.5	0.5
IGKV3-20	21.5	1.2
IGKV3D-11	44.4	7.0
IGKV4-1	19.5	0.9
IGLC6	12.4	0.0
IGLV3-25	34.7	29.6
ITIH1	10.2	0.0
ITIH2	8.2	0.0
ITIH4	6.0	0.0
KLKB1	18.7	0.4
KNG1	13.5	0.0
LRG1	15.5	2.7
LUM	13.8	0.3
ORM1	7.4	0.0
ORM2	10.4	0.0
PON1	10.7	0.0
RBP4	33.8	27.5
SERPINA1	5.6	0.0
SERPINA3	8.1	0.0
SERPINA4	33.6	2.1
SERPINA6	12.9	0.0
SERPINA7	14.0	2.1
SERPINC1	6.7	0.0
SERPIND1	7.6	0.0
SERPINF1	11.2	0.0
SERPINF2	8.5	0.0
SERPING1	10.6	0.0
TF	6.5	0.0
TTN	25.3	26.1
VTN	10.3	0.0

Supplementary Table 3: Associations between proteins and transition status in the overall sample, adjusting for age, sex, and study.

Lower Confidence Interval: LCI; Upper Confidence Interval: UCI; False Discovery Rate: FDR. Results are sorted by P value.

Protein	Odds	LCI	UCI	P value	FDR P value
**C8B**	1.258	1.039	1.523	0.019	0.664
**C4B**	0.809	0.677	0.968	0.021	0.664
**LRG1**	0.81	0.675	0.974	0.025	0.664
**C5**	0.825	0.685	0.994	0.043	0.664
**C4A**	0.835	0.697	1.000	0.051	0.664
**A2M**	1.231	0.998	1.519	0.052	0.664
**FGG**	1.200	0.998	1.442	0.052	0.664
**APOL1**	0.844	0.707	1.007	0.060	0.664
**FGB**	1.192	0.989	1.437	0.065	0.664
**CFB**	0.845	0.705	1.014	0.071	0.664
**FGA**	1.185	0.983	1.429	0.075	0.664
**GC**	0.856	0.71	1.032	0.103	0.664
**VTN**	0.864	0.724	1.032	0.106	0.664
**IGFALS**	0.862	0.719	1.033	0.107	0.664
**IGKV4.1**	1.173	0.965	1.425	0.108	0.664
**C2**	0.862	0.716	1.037	0.114	0.664
**HBB**	0.855	0.703	1.041	0.118	0.664
**RBP4**	0.865	0.718	1.041	0.124	0.664
**HBA1**	0.857	0.704	1.044	0.125	0.664
**PROS1**	0.874	0.732	1.044	0.139	0.674
**CFI**	0.873	0.729	1.046	0.14	0.674
**F12**	1.145	0.949	1.383	0.158	0.7
**AFM**	0.877	0.73	1.053	0.159	0.7
**APOA4**	1.135	0.948	1.359	0.167	0.702
**SERPINA7**	0.89	0.748	1.06	0.191	0.733
**IGHD**	0.887	0.739	1.065	0.199	0.733
**C6**	1.129	0.936	1.362	0.203	0.733
**SERPINA1**	0.893	0.747	1.069	0.218	0.733
**CPN2**	0.892	0.744	1.07	0.218	0.733
**IGHG2**	0.894	0.747	1.071	0.224	0.733
**APCS**	0.892	0.742	1.073	0.225	0.733
**SERPIND1**	0.897	0.749	1.074	0.237	0.749
**HPR**	0.897	0.747	1.078	0.246	0.752
**IGHV5.51**	1.102	0.921	1.319	0.288	0.856
**SERPINC1**	1.101	0.915	1.325	0.309	0.89
**APOC2**	0.918	0.769	1.094	0.339	0.916
**CP**	0.909	0.747	1.106	0.34	0.916
**APOB**	0.92	0.773	1.094	0.345	0.916

**IGLV3-25**	0.919	0.766	1.102	0.361	0.934
**TTN**	0.921	0.77	1.102	0.37	0.934
**SERPING1**	1.083	0.903	1.297	0.39	0.942
**SERPINF2**	0.925	0.775	1.105	0.392	0.942
**APOC3**	1.08	0.901	1.296	0.405	0.942
**C3**	0.929	0.777	1.11	0.416	0.942
**LUM**	1.086	0.888	1.328	0.42	0.942
**IGHV3-30**	1.069	0.893	1.279	0.468	0.961
**HRG**	1.069	0.889	1.286	0.479	0.961
**AHSG**	0.937	0.783	1.122	0.48	0.961
**IGHM**	1.069	0.888	1.285	0.482	0.961
**AZGP1**	1.069	0.881	1.297	0.5	0.961
**GSN**	1.066	0.884	1.287	0.503	0.961
**APOM**	0.939	0.78	1.131	0.507	0.961
**C9**	1.062	0.883	1.277	0.523	0.961
**C8A**	1.059	0.884	1.268	0.535	0.961
**A1BG**	1.057	0.882	1.267	0.546	0.961
**KLKB1**	0.947	0.792	1.134	0.555	0.961
**APOH**	1.054	0.878	1.264	0.575	0.961
**AGT**	0.95	0.781	1.155	0.605	0.961
**IGHG1**	1.048	0.876	1.255	0.608	0.961
**IGKV1.8.9**	0.955	0.798	1.143	0.615	0.961
**IGKV3D.11**	1.048	0.872	1.258	0.618	0.961
**C1R**	0.958	0.799	1.148	0.638	0.961
**SERPINF1**	1.047	0.863	1.27	0.64	0.961
**IGHA1**	1.043	0.868	1.253	0.652	0.961
**C7**	0.959	0.8	1.15	0.652	0.961
**IGHG3**	0.96	0.803	1.147	0.654	0.961
**ITIH4**	1.043	0.863	1.262	0.663	0.961
**APOA1**	1.04	0.867	1.249	0.671	0.961
**APOE**	1.04	0.866	1.249	0.674	0.961
**FN1**	1.041	0.858	1.264	0.681	0.961
**APOD**	1.039	0.865	1.248	0.685	0.961
**ORM2**	0.966	0.804	1.16	0.712	0.98
**APOA2**	0.967	0.806	1.16	0.718	0.98
**TTR**	1.033	0.855	1.247	0.739	0.99
**CFH**	0.971	0.812	1.162	0.75	0.99
**ORM1**	0.972	0.811	1.165	0.757	0.99
**IGHV3.49**	1.027	0.858	1.228	0.772	0.99
**SERPINA4**	0.975	0.812	1.171	0.785	0.99
**C4BPA**	0.978	0.818	1.17	0.808	0.99
**F2**	1.023	0.851	1.229	0.809	0.99
**PON1**	1.021	0.856	1.217	0.819	0.99
**KNG1**	1.02	0.856	1.216	0.826	0.99

**TF**	1.021	0.848	1.229	0.826	0.99
**IGKC**	1.017	0.85	1.218	0.854	0.99
**IGLC6**	1.016	0.848	1.216	0.865	0.99
**IGKV3-20**	1.016	0.847	1.219	0.866	0.99
**ITIH2**	1.014	0.85	1.21	0.877	0.99
**PLG**	1.013	0.844	1.216	0.885	0.99
**IGKV2D.28**	1.011	0.847	1.207	0.906	0.99
**C1S**	1.011	0.84	1.216	0.908	0.99
**IGHG4**	1.01	0.844	1.208	0.914	0.99
**ITIH1**	0.992	0.831	1.184	0.929	0.99
**ALB**	1.008	0.839	1.212	0.931	0.99
**HP**	0.993	0.818	1.206	0.944	0.993
**SERPINA6**	0.996	0.825	1.203	0.968	0.996
**C1QC**	0.997	0.829	1.198	0.971	0.996
**SERPINA3**	1.002	0.838	1.197	0.987	0.996
**CLU**	0.999	0.839	1.191	0.995	0.996
**HPX**	0.999	0.832	1.2	0.996	0.996

Supplementary Table 4: Top ten associations between proteins and transition status in NAPLS2 and NAPLS3, adjusting for age, sex, and study.

Lower Confidence Interval: LCI; Upper Confidence Interval: UCI; False Discovery Rate: FDR.

**Protein**	**Odds**	**LCI**	**UCI**	**P value**	**FDR P Value**
C4B	0.769	0.623	0.948	0.014	0.475
A2M	1.344	1.053	1.714	0.017	0.475
LRG1	0.775	0.623	0.963	0.021	0.475
C4A	0.783	0.635	0.967	0.023	0.475
FGG	1.283	1.028	1.6	0.027	0.475
FGB	1.29	1.027	1.62	0.029	0.475
FGA	1.268	1.018	1.58	0.034	0.48
C2	0.805	0.65	0.997	0.047	0.482
CFB	0.804	0.646	1	0.05	0.482
SERPIND1	0.812	0.658	1.003	0.053	0.482

Supplementary Table 5: Top ten associations between proteins and transition status in NAPLS2 and NAPLS3, adjusting for age, sex, study, and antipsychotic use.

Lower Confidence Interval: LCI; Upper Confidence Interval: UCI; False Discovery Rate: FDR.

Protein	Odds	LCI	UCI	P value	FDR P value
A2M	1.376	1.076	1.759	0.011	0.218
C4B	0.770	0.623	0.952	0.016	0.218
C4A	0.785	0.635	0.970	0.025	0.218
FGB	1.278	1.016	1.608	0.036	0.218
FGA	1.258	1.010	1.567	0.041	0.218
VTN	0.813	0.661	0.999	0.049	0.218
SERPIND1	0.808	0.653	1.000	0.050	0.218
CFB	0.803	0.644	1.001	0.051	0.218
C2	0.810	0.654	1.003	0.053	0.218
C8B	1.211	0.969	1.512	0.092	0.341

Supplementary Table 6: Top ten associations between proteins and transition status in NAPLS3, adjusting for age, sex, study, and BMI.

Lower Confidence Interval: LCI; Upper Confidence Interval: UCI; False Discovery Rate: FDR.

Protein	Odds	LCI	UCI	P value	FDR P Value
C6	1.477	1.029	2.12	0.035	0.931
HRG	1.398	0.98	1.994	0.064	0.931
AGT	0.732	0.497	1.077	0.113	0.931
C8B	1.302	0.926	1.83	0.129	0.931
IGHG3	0.799	0.597	1.069	0.131	0.931
A2M	1.299	0.915	1.843	0.143	0.931
SERPINF1	1.257	0.908	1.742	0.169	0.931
CP	0.791	0.562	1.112	0.178	0.931
FGB	1.254	0.899	1.748	0.183	0.931
FGA	1.25	0.9	1.737	0.183	0.931

Supplementary Table 7: Associations between proteins and Global Assessment of Functioning at 24 months follow-up in NAPLS2 and NAPLS3. Analyses are adjusted for age and sex.

Lower Confidence Interval: LCI; Upper Confidence Interval: UCI; False Discovery Rate: FDR.

Protein	Beta coefficient	LCI	UCI	P-value	FDR P-value
C1R	-1.784	-3.159	-0.408	0.011	0.741
LUM	-1.741	-3.218	-0.263	0.021	0.741
RBP4	1.55	0.145	2.954	0.031	0.741
C1S	-1.482	-2.858	-0.107	0.035	0.741
C6	-1.447	-2.821	-0.074	0.039	0.741
SERPING1	-1.442	-2.85	-0.033	0.045	0.741
TTN	-1.239	-2.575	0.098	0.069	0.979
F2	1.221	-0.177	2.618	0.087	0.996
APOH	1.114	-0.24	2.469	0.106	0.996
IGKV1.8.9	1.075	-0.301	2.451	0.125	0.996
SERPINF1	-1.115	-2.553	0.322	0.128	0.996
CFH	-1.026	-2.377	0.325	0.136	0.996
AGT	1.133	-0.394	2.659	0.145	0.996
C4B	-0.97	-2.363	0.423	0.172	0.996
ORM1	-0.922	-2.29	0.446	0.186	0.996
TF	0.952	-0.463	2.367	0.186	0.996
SERPINF2	-0.914	-2.273	0.445	0.187	0.996
IGHG4	-0.806	-2.127	0.514	0.231	0.996
IGKV3D.11	0.859	-0.584	2.301	0.242	0.996
SERPINA3	-0.829	-2.268	0.61	0.257	0.996
SERPINC1	-0.772	-2.169	0.626	0.278	0.996
IGHG2	-0.771	-2.187	0.645	0.285	0.996
AZGP1	-0.757	-2.153	0.639	0.287	0.996
SERPINA1	0.792	-0.695	2.28	0.294	0.996
APOM	-0.703	-2.035	0.629	0.3	0.996
ORM2	-0.65	-1.989	0.688	0.34	0.996
IGKV4.1	0.641	-0.729	2.012	0.358	0.996
F12	0.621	-0.761	2.004	0.377	0.996
SERPINA6	0.637	-0.792	2.067	0.38	0.996
IGKV3-20	0.629	-0.783	2.042	0.381	0.996
APOE	-0.6	-1.959	0.759	0.386	0.996
GSN	-0.624	-2.067	0.82	0.396	0.996
CFB	-0.589	-1.953	0.774	0.396	0.996
IGLC6	-0.607	-2.034	0.821	0.403	0.996
C3	-0.599	-2.051	0.852	0.417	0.996
IGHV3-30	0.58	-0.85	2.009	0.425	0.996
C5	-0.53	-1.857	0.796	0.432	0.996
CLU	-0.533	-1.885	0.819	0.438	0.996

PON1	-0.525	-1.874	0.825	0.445	0.996
CFI	-0.537	-1.938	0.864	0.451	0.996
ITIH2	-0.5	-1.831	0.832	0.461	0.996
C9	-0.524	-1.942	0.895	0.468	0.996
HRG	-0.5	-1.89	0.891	0.48	0.996
IGHV3.49	-0.475	-1.816	0.867	0.487	0.996
VTN	0.458	-0.919	1.835	0.513	0.996
IGHD	0.422	-0.932	1.776	0.54	0.996
IGFALS	0.425	-0.958	1.808	0.546	0.996
FGA	0.419	-0.971	1.809	0.554	0.996
LRG1	-0.411	-1.797	0.975	0.56	0.996
HP	-0.414	-1.838	1.01	0.568	0.996
APOC3	0.396	-0.98	1.771	0.572	0.996
IGHG3	-0.368	-1.711	0.976	0.591	0.996
HPR	-0.371	-1.734	0.993	0.593	0.996
APCS	0.421	-1.144	1.986	0.596	0.996
SERPINA4	0.365	-1.005	1.736	0.6	0.996
KNG1	0.356	-0.992	1.703	0.604	0.996
C8A	0.361	-1.039	1.762	0.612	0.996
IGKV2D.28	0.341	-1.028	1.709	0.625	0.996
GC	0.335	-1.018	1.687	0.627	0.996
FGG	0.322	-1.049	1.692	0.644	0.996
A2M	-0.373	-1.97	1.224	0.645	0.996
APOC2	0.301	-1.073	1.675	0.667	0.996
ITIH1	0.285	-1.057	1.627	0.676	0.996
APOB	-0.28	-1.63	1.07	0.684	0.996
APOL1	0.277	-1.125	1.678	0.698	0.996
SERPINA7	-0.253	-1.653	1.147	0.722	0.996
C7	-0.24	-1.575	1.095	0.724	0.996
FN1	-0.205	-1.561	1.152	0.767	0.996
IGKC	-0.198	-1.545	1.15	0.773	0.996
FGB	0.197	-1.158	1.551	0.775	0.996
C4BPA	0.203	-1.201	1.608	0.776	0.996
PROS1	-0.193	-1.559	1.172	0.781	0.996
APOA4	-0.202	-1.644	1.239	0.783	0.996
KLKB1	0.195	-1.258	1.649	0.791	0.996
IGLV3-25	0.175	-1.166	1.515	0.798	0.996
CP	0.186	-1.251	1.624	0.799	0.996
CPN2	-0.164	-1.555	1.227	0.817	0.996
AFM	0.145	-1.216	1.506	0.834	0.996
C2	-0.151	-1.593	1.291	0.836	0.996
HPX	-0.136	-1.471	1.2	0.842	0.996
A1BG	-0.143	-1.559	1.273	0.843	0.996
ITIH4	-0.138	-1.642	1.365	0.856	0.996

SERPIND1	-0.104	-1.506	1.298	0.884	0.996
HBA1	-0.093	-1.531	1.344	0.898	0.996
IGHA1	0.085	-1.298	1.467	0.904	0.996
PLG	-0.069	-1.421	1.284	0.921	0.996
C1QC	-0.068	-1.465	1.329	0.924	0.996
IGHV5.51	-0.07	-1.55	1.411	0.926	0.996
ALB	-0.063	-1.499	1.372	0.931	0.996
APOA2	-0.05	-1.447	1.347	0.944	0.996
APOA1	-0.042	-1.494	1.409	0.954	0.996
HBB	-0.038	-1.466	1.389	0.958	0.996
C8B	-0.038	-1.562	1.485	0.96	0.996
APOD	0.024	-1.39	1.438	0.973	0.996
IGHM	0.018	-1.492	1.528	0.981	0.996
TTR	-0.017	-1.461	1.427	0.981	0.996
IGHG1	-0.015	-1.39	1.359	0.983	0.996
C4A	0.004	-1.372	1.38	0.995	0.996
AHSG	-0.003	-1.343	1.337	0.996	0.996

Supplementary Table 8: Top ten associations between proteins and transition status in NEURAPRO, adjusting for age, sex, and BMI.

Standard Error: SE; Lower Confidence Interval: LCI; Upper Confidence Interval: UCI; False Discovery Rate: FDR.

Protein	Odds	LCI	UCI	P value	FDR P value
**C5**	0.658	0.443	0.976	0.038	0.97
**AZGP1**	1.52	0.999	2.314	0.051	0.97
**IGHD**	0.696	0.476	1.016	0.06	0.97
**IGHV5.51**	1.445	0.977	2.137	0.065	0.97
**IGKV4.1**	1.495	0.962	2.324	0.074	0.97
**C8B**	1.432	0.95	2.157	0.086	0.97
**SERPINA7**	0.753	0.535	1.06	0.104	0.97
**IGKV3D.11**	1.388	0.923	2.089	0.116	0.97
**APOH**	1.401	0.914	2.147	0.122	0.97
**IGHG2**	0.749	0.515	1.088	0.129	0.97

Supplementary Table 9: Top ten associations between proteins and Social and Occupational Functioning at 24 months follow-up in NEURAPRO. Analyses are adjusted for age, sex and BMI.

Lower Confidence Interval: LCI; Upper Confidence Interval: UCI; False Discovery Rate: FDR.

Protein	Beta coefficient	LCI	UCI	P value	FDR P value
**F12**	-1.727	-3.918	0.464	0.122	1
**APOB**	1.615	-0.553	3.783	0.143	1
**C1R**	-1.462	-3.531	0.607	0.165	1
**TTN**	1.459	-0.675	3.593	0.179	1
**APOM**	1.47	-0.681	3.621	0.179	1
**APOC2**	1.461	-0.679	3.600	0.179	1
**KNG1**	-1.331	-3.371	0.709	0.200	1
**FGA**	-1.291	-3.464	0.882	0.243	1
**RBP4**	1.23	-0.895	3.355	0.255	1
**HPR**	-1.171	-3.257	0.915	0.270	1

Supplementary Table 10: Spearman's rho correlation between proteins measured with mass spectrometry and multiplex immunoassay (Myriad Rules Based Medicine, Human Discovery Map).

Protein	Spearman's Rho
**A2M**	0.50
**HP**	0.60
**IGHM**	0.64
**C3**	0.50
**TTR**	0.40
**CLU**	0.21
**PROS1**	0.13

Supplementary Figures

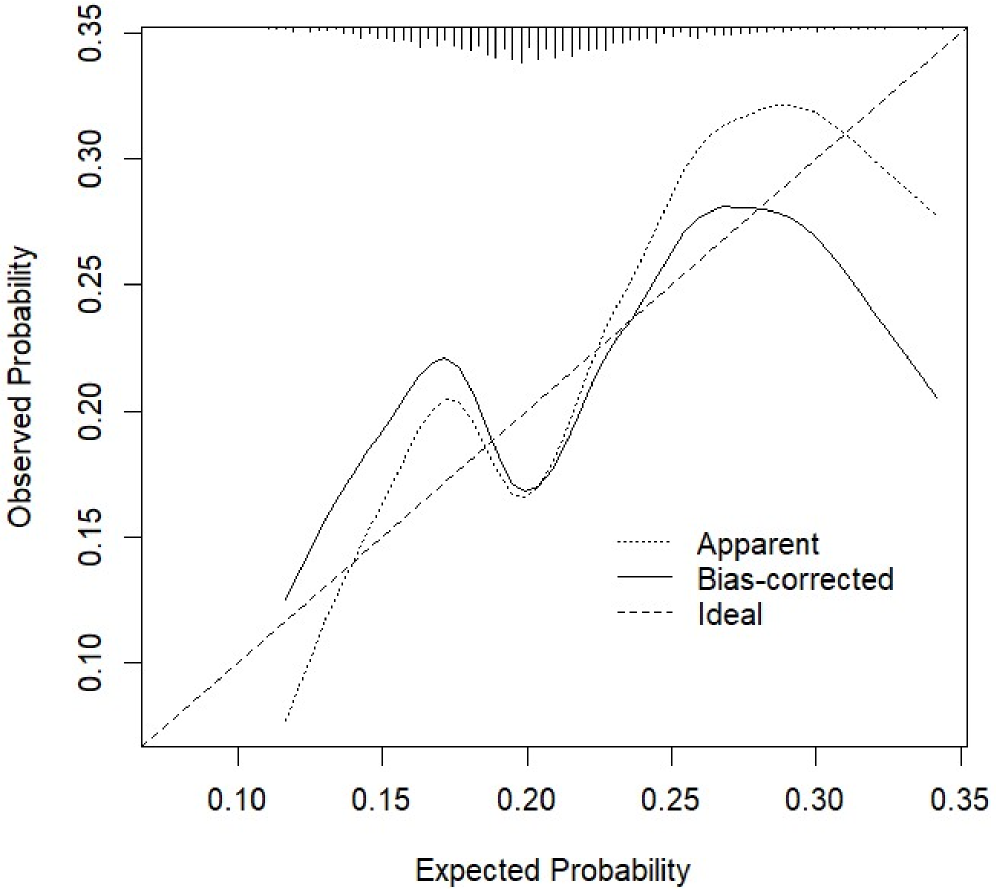

Supplementary Figure 1: Calibration plot for the *a-priori* specified model of transition, comparing observed probabilities and expected (predicted) probabilities.

References

1. Miller TJ, McGlashan TH, Rosen JL, et al. Prodromal assessment with the structured interview for prodromal syndromes and the scale of prodromal symptoms: predictive validity, interrater reliability, and training to reliability. *Schizophrenia bulletin*. 2003;29(4):703-715.

2. Hall RC. Global assessment of functioning: a modified scale. *Psychosomatics*. 1995;36(3):267-275.

3. Nelson B, Amminger GP, Yuen HP, et al. NEURAPRO: a multi-centre RCT of omega-3 polyunsaturated fatty acids versus placebo in young people at ultra-high risk of psychotic disorders—medium-term follow-up and clinical course. *npj Schizophrenia*. 2018;4(1):11. doi:10.1038/s41537-018-0052-x

4. Morosini PL, Magliano L, Brambilla L al, Ugolini S, Pioli R. Development, reliability and acceptability of a new version of the DSM-IV Social and Occupational Functioning Assessment Scale (SOFAS) to assess routine social funtioning. *Acta Psychiatrica Scandinavica*. 2000;101(4):323-329.

5. Bache N, Geyer PE, Bekker-Jensen DB, et al. A Novel LC System Embeds Analytes in Pre-formed Gradients for Rapid, Ultra-robust Proteomics. *Mol Cell Proteomics*. 2018;17(11):2284-2296. doi:10.1074/mcp.tir118.000853

6. Meier F, Brunner AD, Koch S, et al. Online Parallel Accumulation–Serial Fragmentation (PASEF) with a Novel Trapped Ion Mobility Mass Spectrometer*. *Molecular & Cellular Proteomics*. 2018;17(12):2534-2545. doi:10.1074/mcp.TIR118.000900

7. Cox J, Hein MY, Luber CA, Paron I, Nagaraj N, Mann M. Accurate Proteome-wide Label-free Quantification by Delayed Normalization and Maximal Peptide Ratio Extraction, Termed MaxLFQ *. *Molecular & Cellular Proteomics*. 2014;13(9):2513-2526. doi:10.1074/mcp.M113.031591

8. Riley RD, Snell KI, Ensor J, et al. Minimum sample size for developing a multivariable prediction model: PART II - binary and time-to-event outcomes. *Statistics in Medicine*. 2019;38(7):1276-1296. doi:10.1002/sim.7992

9. Moons KG, Wolff RF, Riley RD, et al. PROBAST: a tool to assess risk of bias and applicability of prediction model studies: explanation and elaboration. *Annals of internal medicine*. 2019;170(1):W1-W33.

10. Steyerberg EW, Harrell FE, Borsboom GJJM, Eijkemans MJC, Vergouwe Y, Habbema JDF. Internal validation of predictive models: Efficiency of some procedures for logistic regression analysis. *Journal of Clinical Epidemiology*. 2001;54(8):774-781. doi:10.1016/S0895-4356(01)00341-9

11. Noma H, Shinozaki T, Iba K, Teramukai S, Furukawa TA. Confidence intervals of prediction accuracy measures for multivariable prediction models based on the bootstrap-based optimism correction methods. *Statistics in Medicine*. 2021;40(26):5691-5701. doi:10.1002/sim.9148
